# Paradoxical Eczema Associated With Interleukin-17A Inhibitor Use in a Patient With Generalized Pustular Psoriasis Accompanied by Asthma

**DOI:** 10.7759/cureus.64680

**Published:** 2024-07-16

**Authors:** Kazuki Yatsuzuka, Jun Muto, Ken Shiraishi, Yasuhiro Fujisawa, Masamoto Murakami

**Affiliations:** 1 Department of Dermatology, Ehime University Graduate School of Medicine, Toon, JPN

**Keywords:** generalized pustular psoriasis, psoriasis, interleukin-17 inhibitor, eczema, paradoxical eczema

## Abstract

Although rare, paradoxical eczema (PE) is an adverse event associated with the use of biological agents to treat psoriasis, particularly in patients with atopic predispositions. We report the first case of severe PE induced by secukinumab in a patient with generalized pustular psoriasis (GPP) and asthma. A woman in her 50s with a history of interstitial nephritis attributable to Sjogren’s syndrome experienced a flare-up of GPP after discontinuing mycophenolate mofetil and was hospitalized. Treatment with secukinumab accompanied by an increased prednisolone level afforded rapid improvement, but she subsequently developed widespread, itchy, serous papules and erythema. A biopsy confirmed that the erythema was an eczematous reaction, thus PE. Her condition improved after switching from secukinumab to deucravacitinib with a temporary increase in the prednisolone level; no recurrence of GPP or PE was observed for 11 months. Elevated serum levels of interleukin (IL)-17A, IL-22, and the Th2 chemokine TARC recorded at the onset time of PE suggested that these mediators contributed to the observed pathology. Our case highlights the need for careful consideration when prescribing IL-17 inhibitors to patients with GPP, particularly those with atopic predispositions, given the potential activation of the Th2 axis and thus severe eczematous reactions. Further research is required to understand the essential nature of PE in patients with GPP and the roles of IL-17A and IL-22 in this context.

## Introduction

Paradoxical eczema (PE) is a rare adverse event associated with the use of biological agents to treat patients with psoriasis. The reported incidence ranges from 1.0% to 12.1% [1]. Most reported cases are plaque psoriasis, and reports of generalized pustular psoriasis (GPP) patients are scarce. In severe cases, drug discontinuation or a change in treatment may be necessary. Atopic predisposition (hay fever, asthma, or atopic dermatitis) has previously been reported to be the most important factor associated with PE development [1]. In a recent prospective cohort study of 13,699 psoriasis patients, interleukin (IL)-17 inhibitors were the most common biologics associated with PE [2].

We herein report a GPP patient with severe PE induced by secukinumab. We analyzed cytokine dynamics in serum and reported the possible involvement of IL-22 in PE pathology. The study was approved by the Ethics Committee of Ehime University (approval number: 1808003). She gave us written informed consent for publication, and her anonymity was preserved using methods approved by the Ethics Committee.

## Case presentation

**Figure 1:**
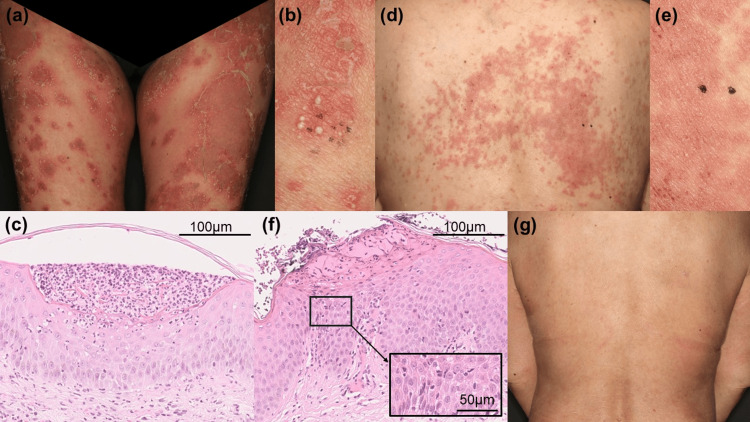
Cutaneous and histological findings At admission, our patient presented with multiple painful erythema on the extremities and trunk, accompanied by pustules around the edges (A). A biopsy of a pustule on the right forearm (B) revealed that this was a Kogoj spongiform pustule (hematoxylin-and-eosin staining) (C). Four months after discharge, the patient developed itchy serous papules, crusts, and exudative erythema on the extremities and trunk (D). A biopsy of the back erythema (E) revealed crusts, epidermal thickening, spongiosis, and lymphocyte infiltration into the epidermis (hematoxylin-and-eosin staining) (F). After discontinuation of secukinumab and an increase in the prednisolone dose, the rash improved in about two weeks (G).

**Figure 2:**
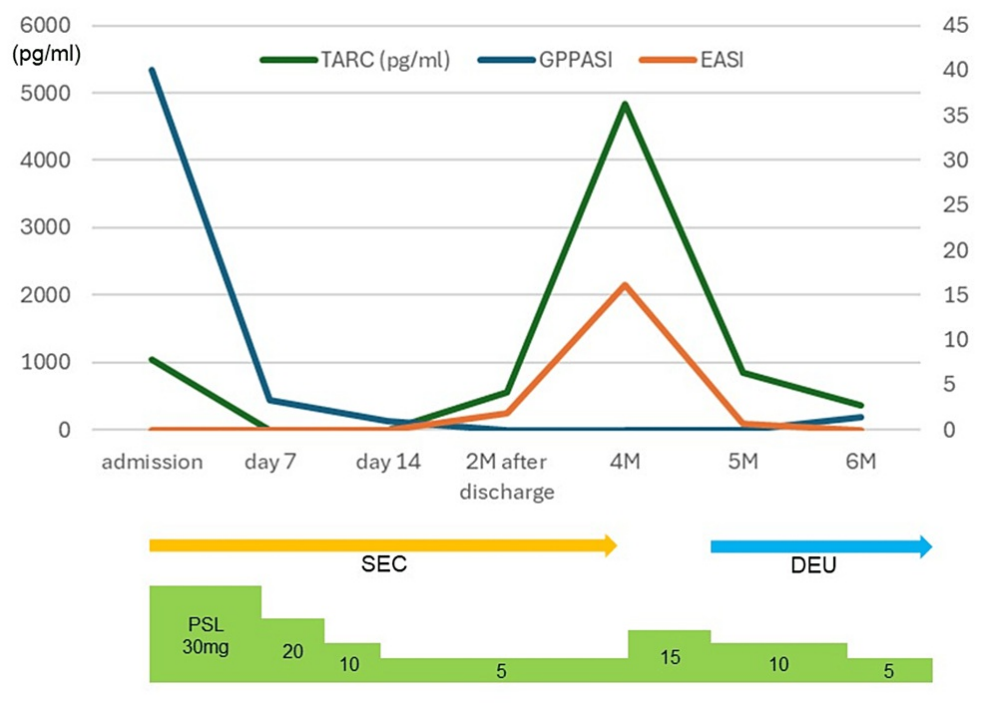
Clinical course after a GPP flare-up GPPASI: Generalized Pustular Psoriasis Area and Severity Index, EASI: Eczema Area and Severity Index, SEC: secukinumab, DEU: deucravacitinib, PSL: prednisolone, GPP: generalized pustular psoriasis

A woman in her 50s with a history of asthma and interstitial nephritis attributable to Sjogren’s syndrome was taking 5 mg/day prednisolone and 1,000 mg/day mycophenolate mofetil (MMF). She also had GPP and had been treated with steroid ointments and oral etretinate for 15 years; the GPP was in near remission. However, due to a COVID-19 infection, she had to discontinue MMF; soon thereafter, she experienced a GPP flare-up and was admitted to our department. Upon admission, she exhibited a fever of 37.9°C and edema in both lower limbs. Multiple erythema with pustules around the edges of the lesions were present on the extremities and trunk, accompanied by pain (Figure 1A). The Generalized Pustular Psoriasis Area and Severity Index (GPPASI) was 40.1. Laboratory tests revealed elevated levels of white blood cells (18,200/μL) with neutrophilia (17,108/μL), elevated serum levels of C-reactive protein (4.42 mg/dL), and hypoalbuminemia (2.9 g/dL). The TARC level was also elevated (1,041 pg/mL). A biopsy of a pustule from the right forearm (Figure 1B) identified it as the Kogoj spongiform type (Figure 1C). Together, the data indicated a GPP flare-up with systemic symptoms. We did not conduct mutation analysis of the IL-36RN, CARD14, AP1S3, and MPO genes because we could not obtain the patient’s consent. We commenced secukinumab at 300 mg/week and increased prednisolone to 30 mg/day. The fever rapidly subsided, and the limb pain, erythema, and pustules improved. The GPPASI improved from 40.1 to 0.9 within two weeks of admission, and the patient was discharged in week 3. She continued to take 300 mg/month of secukinumab and 5 mg/day of prednisolone on an outpatient basis; her condition was well-controlled. However, about two months after discharge, she developed itchy serous papules and erythema that tended to coalesce on the extremities and trunk and gradually expanded. Despite treatment with topical steroids and oral antihistamines, no improvement was observed. The condition rapidly worsened after further secukinumab administration; exudates and crusts developed (Figure 1D). As there were no pustules in the erythema and as the TARC level was markedly elevated (4,842 pg/mL), we suspected PE to be attributable to secukinumab. A biopsy of the back (Figure 1E) showed crusts, epidermal thickening, spongiosis, and lymphocyte infiltration into the epidermis, indicating an eczematous reaction (Figure 1F). Four months after discharge, secukinumab was discontinued, and prednisolone was increased to 15 mg/day. The rash improved within two weeks (Figure 1G), and the TARC level markedly dropped; prednisolone was reduced to 10 mg/day, and 6 mg/day of deucravacitinib was initiated as an alternative to secukinumab (Figure 2).

Since that time, she has not experienced a GPP or PE recurrence for 11 months. Prednisolone continues at a maintenance dose of 5 mg/day.

**Figure 3:**
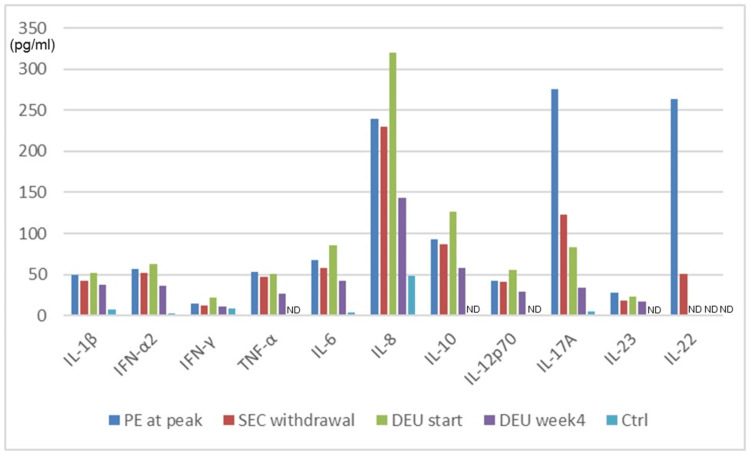
Serum levels of various cytokines as measured via LEGENDplex and ELISA at different times Data are presented as means of two assays. ND: not detected, PE: paradoxical eczema, SEC: secukinumab, DEU: deucravacitinib, Ctrl: control (normal volunteer)

After PE onset, the serum levels of IL-4, IL-5, IL-13, IL-22, and interferon (IFN)-β were measured via ELISA, and those of IL-1β, IFN-α2, IFN-γ, tumor necrosis factor (TNF)-α, IL-6, IL-8, IL-10, IL-12p70, IL-17A, and IL-23 were assayed employing the LEGENDplex system (BioLegend, San Diego, CA, USA). IL-4, IL-5, IL-13, and IFN-β levels did not increase at any time after PE onset (data not shown), but the levels of IL-17A and IL-22 were elevated at the time of the PE peak and tended to decrease as PE improved (Figure 3).

## Discussion

The mechanism by which PE develops in patients with plaque psoriasis remains unclear. Previous cytokine analyses suggest that blocking of the Th1/Th17 axis activates the Th2 axis of lesions and/or serum in patients with genetic predispositions [3,4] toward the development of secondary eczematous reactions. To the best of our knowledge, although one mild case of PE has been reported in a GPP patient [5], no severe PE in a GPP patient has yet been described, perhaps because GPP is rarer than plaque-type psoriasis. Interestingly, a previous study found that serum levels of TARC were significantly higher in GPP patients than in those with plaque psoriasis [6], suggesting that GPP may be associated with both Th2 and Th17 activation. In our case, the levels were slightly elevated at the time of the GPP recurrence. In addition, our patient had a history of asthma, a known risk factor for PE development, perhaps indicating a susceptibility to Th2 axis skewing. Indeed, the level of TARC, a Th2 chemokine, significantly increased after secukinumab initiation. In Western countries, few biological agents are approved for GPP treatment. By contrast, many biologics have been approved in Japan. A nationwide retrospective cohort study using the Japanese national inpatient database found that the use of IL-17 inhibitors has increased in recent years. The treatment outcomes of IL-17 inhibitors are comparable to those of TNF-α inhibitors [7]. On the other hand, their use is most associated with PE development [2]. Therefore, if a GPP patient exhibits an atopic predisposition, as in our case, careful consideration is required when initiating IL-17 inhibitors. However, the detailed pathogenesis of PE remains largely unknown, and further investigations are required.

Although TARC levels increased at the time of PE onset (Figure 2), the levels of Th2 cytokines (IL-4, IL-5, and IL-13) did not. However, IL-22 levels peaked at PE onset and tended to decrease as PE improved (Figure 3). One report on the PE lesions of plaque psoriasis patients caused by IL-17 inhibitors suggested that IL-22 plays a significant role in PE development [8]. Another study indicated that IL-22 increases TARC production by HaCaT cells [9]. Together, the data suggest that IL-22 might elevate TARC levels in PE patients when IL-22 expression is induced by IL-17 inhibitors, as in our case. As we prescribed deucravacitinib only after the PE had improved, we cannot evaluate its effectiveness, but it suppresses IL-22 synthesis [10]. Hence, deucravacitinib may be a useful treatment option for PE caused by IL-17 inhibitors. Notably, IL-17A levels increased despite the use of IL-17 inhibitors (Figure 3). Li et al. [11] also reported increased IL-17A levels in four plaque psoriasis patients with PE caused by secukinumab. However, further exploration of the underlying mechanism is required.

## Conclusions

To the best of our knowledge, this is the first report of a patient with GPP and asthma who presented with severe PE induced by IL-17 inhibitors. Atopic predisposition should be considered when deciding on what biologics to use to treat patients with plaque psoriasis and also those with GPP. Cytokine dynamics in the patient's serum suggest the involvement of IL-22 in the development of PE, as in plaque psoriasis. Further research is required to understand the essential nature of PE in patients with GPP.
